# An essential role of a ferritin-like protein in acid stress tolerance of *Listeria monocytogenes*

**DOI:** 10.1007/s00203-014-1053-4

**Published:** 2014-10-29

**Authors:** Dorota Milecka, Anna Samluk, Katarzyna Wasiak, Agata Krawczyk-Balska

**Affiliations:** Department of Applied Microbiology, Faculty of Biology, University of Warsaw, Miecznikowa 1, 02-096 Warsaw, Poland

**Keywords:** *Listeria monocytogenes*, Acid stress, Ferritin-like protein

## Abstract

The expression of ten genes of *Listeria monocytogenes* previously identified as penicillin G-inducible was transcriptionally analyzed in the presence of 0.5 M KCl, pH 5.0 and 42 °C. This study revealed that all the genes are upregulated by osmotic stress, seven by acid stress and four by temperature stress conditions. The contribution of a gene encoding a ferritin-like protein (*fri*), a two-component phosphate-response regulator (*phoP*) and an AraC/XylS family transcription regulator (*axyR*) to temperature, acid and osmotic stress tolerance was further examined by analysis of nonpolar deletion mutants. This revealed that a lack of PhoP or AxyR does not affect the ability to grow under the tested stress conditions. However, the **Δ** *fri* strain showed slightly delayed growth under osmotic and clearly impaired growth under acid stress conditions, indicating an important role of the ferritin-like protein in acid stress tolerance.

## Introduction

*Listeria monocytogenes* is a foodborne Gram-positive pathogen that causes rare but severe disease in humans and animals. This bacterium can adapt to survive in different environmental conditions including a wide range of temperatures (0–45 °C), relatively low pH values, high concentrations of salt and the presence of a high concentration of β-lactam antibiotics that are antibiotics of choice in the treatment of listeriosis. The adaptive abilities allow the wide distribution of *L. monocytogenes* in the natural environment, survival of food processing and rapidly changing conditions encountered during gastrointestinal passage and subsequent stages of infection and antibiotic therapy (Vazquez-Boland et al. 2001; Hof 2003; Gandhi and Chikindas 2007). Taking into account the importance of the adaptive abilities of *L. monocytogenes*, the primary goals of much research effort focus on identifying genes playing an important role in stress tolerance. So far, the best documented role in the response of *L. monocytogenes* to stressful conditions has been established for genes coding for sigma factors and two-component signal transduction systems that have highlighted the importance of regulator proteins in the adaptation of *L. monocytogenes* (Ferreira et al. 2001; Williams et al. 2005; Raimann et al. 2009). What is worth note is that individual regulator proteins and sigma factors often play an important role in response to more than one stress factor (Cotter et al. 1999, 2002; Ferreira et al. 2001; Kallipolitis et al. 2003; Begley et al. 2006; Raimann et al. 2009). The characterization of the variation of *L. monocytogenes* gene expression patterns in different harmful conditions has also been the subject of intensive study since it could help to elucidate the mechanisms of *L. monocytogenes* tolerance to different stresses. Interestingly, a recent transcriptomic study showed large overlap between the cefuroxime stimulon and genes known to be induced in *L. monocytogenes* in blood and during intracellular infection which suggests that the pattern of transcriptional changes related to cell-envelope-stress response overlaps in different stress conditions (Nielsen et al. 2012). Recently, we identified ten genes of *L. monocytogenes* whose expression was upregulated in response to penicillin G pressure (Krawczyk-Balska et al. 2012). Two of these, namely *phoP* and *axyR*, encode regulatory proteins. Gene *phoP* encodes a two-component phosphate-response regulator, and *axyR* gene encodes a transcriptional regulator from the AraC/XylS regulator family (Williams et al. 2005; Sabet et al. 2008). Among the identified genes was also *fri* encoding an iron-binding ferritin-like protein (Fri) that belongs to the Dps (*D*NA-binding *p*roteins from *s*tarved cells) family of proteins (Haikarainen and Papageorgiou 2010). Fri of *L. monocytogenes* is not a sensu stricto regulator protein. However, it contributes to virulence and plays a role in protection against multiple stresses. Furthermore, in the *fri* mutant strain, significant changes in the content of the global regulators catabolite control protein A (CcpA) and anti-sigma B factor (RsbW) were observed, thus suggesting that Fri has a global impact on the *L. monocytogenes* regulatory network (Dussurget et al. 2005; Olsen et al. 2005). Susceptibility studies showed that Fri is a mediator of β-lactam tolerance and innate resistance to cephalosporins, and that the protein plays a critical role in the control of *L. monocytogenes* cell envelope structure and stability under β-lactam pressure, whereas PhoP and AxyR do not influence susceptibility and tolerance to this class of antibiotics (Krawczyk-Balska et al. 2012; Krawczyk-Balska and Lipiak 2013).

The aim of the present study was to determine how the transcription of previously identified penicillin G-inducible genes changes under temperature, acid and osmotic pressure and to examine whether *fri*, *phoP* and *axyR* genes play a role in the adaptation of *L. monocytogenes* to these stress conditions.

## Materials and methods

### Bacterial strains, media, plasmids and DNA techniques

The *L. monocytogenes* EGD (serotype 1/2a) wild-type strain was kindly provided by S.J. Foster, University of Sheffield, United Kingdom. Isogenic EGDΔ*phoP* and EGDΔ*axyR* deletion mutants were constructed in a previous study (Krawczyk-Balska et al. 2012), and the isogenic EGDΔ*fri* deletion mutant was a generous gift from Hanne Ingmer, Royal Veterinary and Agricultural University, Denmark. *L. monocytogenes* strains were grown in brain heart infusion (BHI) broth medium (Oxoid).

### Total RNA isolation

For RNA isolation, a culture of wild-type *L. monocytogenes* EGD was inoculated and incubated overnight at 37 °C. The following morning, the culture was diluted 1:50 into fresh medium. The culture was grown at 37 °C with aeration to an OD_600_ of 0.2. At this point, for osmotic stress and acid stress treatments, the cells were harvested by centrifugation and then resuspended in BHI broth containing 0.5 M KCl or resuspended in BHI broth adjusted to pH 5 with HCl, respectively, and incubation was continued for 90 min at 37 °C. For temperature stress treatment, once the refreshed culture attained an OD_600_ of 0.2, the culture was shifted to 42 °C for 90 min. As the control, the unstressed culture was used, and once it reached an OD_600_ of 0.2 it was grown for an additional 90 min at 37 °C.

Total RNA was isolated using the hot acid phenol procedure as described previously (McGrath et al. 2001). Contaminating DNA was degraded using RNase-free DNase (Fermentas). The concentration and purity of the RNA preparations was then estimated by measuring the A260 and A280 with a NanoDrop ND-1000 spectrophotometer. The RNA quality and integrity was further analyzed by agarose gel electrophoresis. The absence of DNA from RNA preparations was verified by the failure to amplify a 16S rRNA gene fragment in a 30-cycle PCR using 1 μg of RNA as the template. The prepared RNA was stored at −70 °C until required for analysis.

### Transcriptional analysis of the studied genes

To compare the level of transcription of the identified genes in nonstressed cells and in cells growing in different stress condition, reverse transcriptase PCR (RT-PCR) was performed as described previously using the primers specific for the identified genes and for the 16S rRNA gene (Krawczyk-Balska et al. 2012). The RT-PCR products were quantified by densitometric analysis of DNA bands on gel images using ImageQuant™ TL software (GE Healthcare, UK).

### Growth in stress conditions

To examine the growth of *L. monocytogenes* strains under osmotic or acid stress conditions, overnight cultures were diluted (1:100) in BHI broth containing 0.5 M KCl or HCl (pH 5), respectively, and incubated with shaking at 37 °C. To examine the growth under temperature stress conditions, overnight cultures were diluted (1:100) in BHI broth and incubated with shaking at 42 °C. Cell growth was monitored spectrophotometrically by determining the OD_600_.

## Results and discussion

**Fig. 1:**
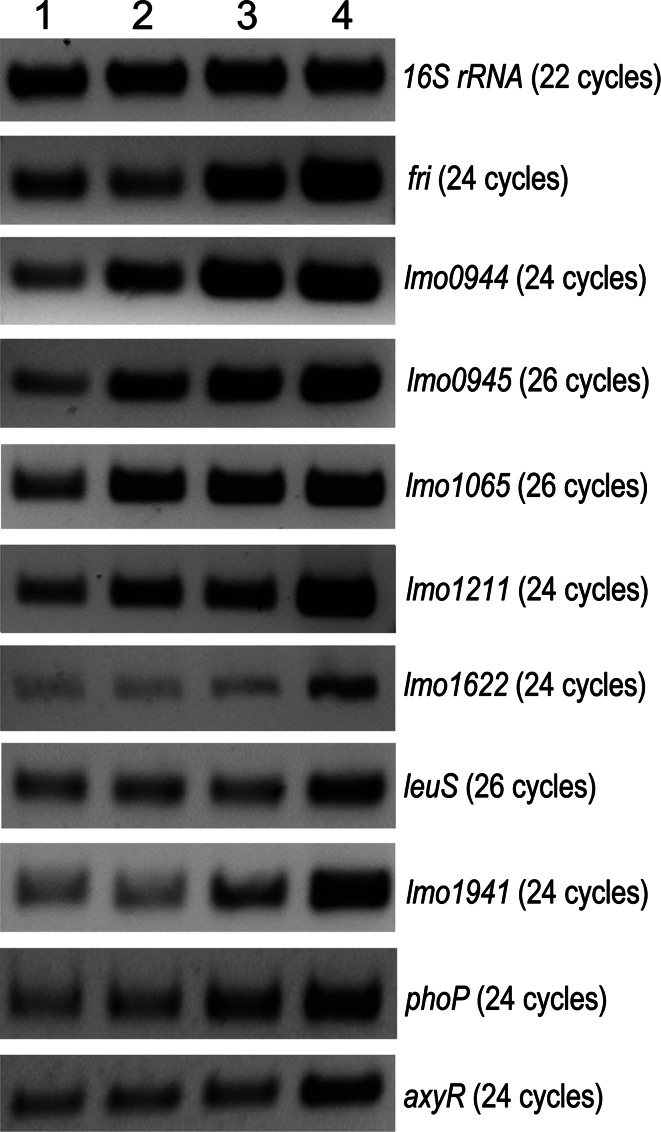
Transcriptional analysis of gene expression under temperature, acid and osmotic stress conditions using RT-PCR. Total RNA was isolated from exponential phase cultures of *L. monocytogenes* EGD strain grown in BHI at 37 °C (*1*) and exposed to 42 °C (*2*) or pH 5 (*3*) or 0.5 M KCl (*4*) for 90 min in each case. All RT-PCRs were performed three times from three separate RNA preparations. In all cases, control PCR were performed to ensure the complete removal of DNA from RNA preparations prior to reverse transcription

Recently, we identified several genes of *L. monocytogenes* whose expression was upregulated in the presence of 0.09 µg/ml penicillin G, which equates to 0.75 of the MIC (minimal inhibitory concentration) value. To examine whether the expression of the identified genes was specifically induced by penicillin G, or if it was also induced by other stresses, transcriptional analysis of these genes was performed under temperature, acid and osmotic stress conditions (Fig. 1), and their relative expression levels were quantified (Table 1). This analysis revealed that all of the selected genes were induced under osmotic stress conditions. The transcription of seven of them, namely *fri*, *lmo0944*, *lmo0945*, *lmo1065*, *lmo1211*, *lmo1941*, *phoP*, was upregulated under acid stress conditions. Four of the genes, namely *lmo0944*, *lmo0945*, *lmo1065* and *lmo1211*, were upregulated under temperature stress conditions; thus, these genes exhibited an elevated level of expression under all of the stress conditions tested. Transcriptional analysis revealed that the upregulation of expression of the studied genes is not restricted to cell wall-acting antibiotic pressure, indicating that these genes belong to a more general stress stimulon of *L. monocytogenes.* Similar results concerning the cellular response of *Streptomyces coelicor* to antibiotics that target the cell envelope have been reported (Hesketh et al. 2011). This study clearly showed that antibiotic treatment induces changes in the expression of many genes of the heat, osmotic and oxidative stress regulons. Strikingly, in our study, the expression of all penicillin G-inducible genes was also upregulated in response to osmotic stress conditions. This finding suggests that the cell-envelope-stress response in *L. monocytogenes* is linked to the osmotic stress response. This observation is consistent with the results of a study on the cefuroxime stimulon of *L. monocytogenes* that demonstrated that the expression of highly cefuroxime-induced genes is also induced by osmotic stress (Nielsen et al. 2012).

**Table 1:** Relative expression levels of the studied *L. monocytogenes* genes

Gene	Fold change in expression^a^		
	42 °C	pH 5	0.5 M KCl
*fri*	0.9 ± 0.1	2.7 ± 0.6*	3.7 ± 1.0*
*lmo944*	2.9 ± 0.1**	4.8 ± 0.7**	4.8 ± 0.9*
*lmo945*	3.0 ± 0.6*	3.2 ± 0.5*	3.9 ± 0.6*
*lmo1065*	3.2 ± 0.5*	3.1 ± 0.3**	4.7 ± 0.8*
*lmo1211*	2.4 ± 0.4*	2.3 ± 0.4*	4.7 ± 0.6*
*lmo1622*	1.2 ± 0.3	1.3 ± 0.3	5.1 ± 0.4**
*leuS*	1.2 ± 0.2	1.3 ± 0.3	3.4 ± 0.9*
*lmo1941*	1.1 ± 0.2	2.3 ± 0.3*	5.1 ± 0.2**
*phoP*	1.5 ± 0.4	2.3 ± 0.5*	5.0 ± 0.5**
*axyR*	1.2 ± 0.3	1.3 ± 0.2	3.7 ± 0.6*

^a^The numbers given are the relative amounts of the RT-PCR products obtained using a template of total RNA isolated from *L. monocytogenes* EGD exposed to 42 °C, pH 5 or 0.5 M KCl, in comparison with the corresponding amounts for the wild-type strain grown in BHI broth at 37 °C without any stressors. The RT-PCR products were quantified by measuring the level of band fluorescence using ImageQuant software. Relative expression levels were normalized to the levels of 16S rRNA. Asterisks indicate significant differences according to Student’s *t*-test (*P < 0.05; **P < 0.01)

**Fig. 2:**
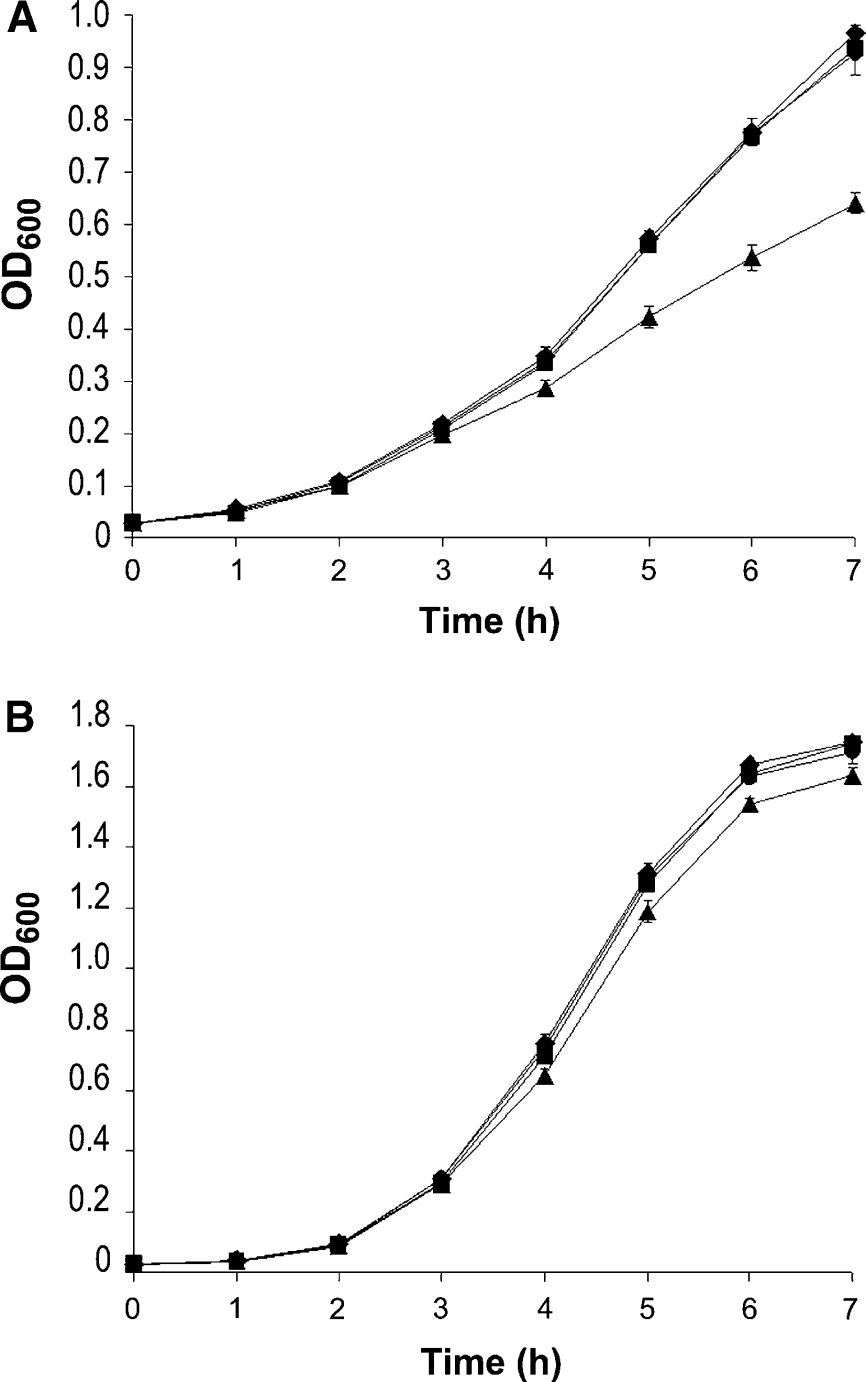
Growth of wild-type *L. monocytogenes* EGD (*filled circle*), Δ*axyR* mutant (*filled diagonal*), Δ*phoP* mutant (*filled square*) and Δ*fri* mutant (*filled triangle*) in BHI broth pH 5 adjusted with HCl (**a**) and in BHI broth supplemented with 0.5 M KCl (**b**). Overnight cultures were inoculated (1:100) into BHI broth supplemented with appropriate stressor, and cultures were incubated with shaking at 37 °C. Cell growth was measured spectrophotometrically by determining the optical density at 600 nm. *Error bars*, standard deviations from three independent experiments

Given the significant role of regulatory proteins in *L. monocytogenes* response to multiple stresses, the genes *fri*, *phoP* and *axyR* were chosen for further investigation concerning their involvement in temperature, acid and osmotic stress tolerance since these genes encode proteins with regulatory potential. For this purpose, the wild-type and ∆*phoP*, ∆*axyR* and ∆*fri* mutants were analyzed for their responses to temperature, acid and osmotic stress. No difference in growth rates was observed when the strains were cultured at 42 °C (data not shown). Likewise, the ∆*phoP* and ∆*axyR* mutants and the wild-type grew equally well in media adjusted to pH 5.0 and in media supplemented with 0.5 M KCl (Fig. 2). Thus, the analysis revealed that the lack of *phoP* and *axyR* does not affect the adaptation of *L. monocytogenes* to temperature, acid or osmotic stress conditions. However, the growth rate of the Δ*fri* mutant strain was slightly reduced in comparison with that of the wild-type strain in a hyperosmotic environment, and growth of the ∆*fri* mutant was clearly impaired under acid stress conditions (Fig. 2). Thus, the Fri protein of *L. monocytogenes* was found to be involved to some extent in osmotic stress tolerance although it appears to play a minor role under these stress conditions. Similarly, increased expression of other members of the Dps family, such as Dps of *E. coli*, DpsA of *B. subtilis* and MsDps1 of *Mycobacterium smegmatis*, has been observed under osmotic stress conditions, which suggests the participation of these proteins in adaptation to this stress (Antelmann et al. 1997; Weber et al. 2006; Chowdhury et al. 2007), but so far they have not been shown to play a key role in osmotic stress tolerance. Interestingly, the listerial ferritin was found to play an essential role in acid stress tolerance of this bacterium. This observation is consistent with reports that mutants of *E. coli* lacking the Dps protein are much more susceptible to DNA strand breaks than the wild type under acidic conditions, which results in a sharp decline in dps mutant survival (Choi et al. 2000; Jeong et al. 2008).
